# The Invasive Management of Pain: Diagnosis and New Treatment Options

**DOI:** 10.7759/cureus.42717

**Published:** 2023-07-31

**Authors:** Victor Mayoral Rojals, Cesar Amescua Garcia, Pasquale Denegri, Marco Antonio Narvaez Tamayo, Giustino Varrassi

**Affiliations:** 1 Department of Anesthesia, Belvitge Hospital, L'Hospitalet de Llobregat, ESP; 2 Research Department, Hospital Angeles, Tijuana, MEX; 3 Anesthesia, Intensive Care, and Pain Medicine, Sant'Anna and San Sebastiano Hospital, Caserta, ITA; 4 Interventional Pain Medicine and Palliative Care, Hospital Obrero N. 1-HM1, La Paz, BOL; 5 Pain Medicine, Paolo Procacci Foundation, Rome, ITA

**Keywords:** spinal neuromodulation, ultrasound, complex regional pain syndrome, lumbar spinal stenosis, neurogenic claudication, annular fissure, bulging discs, degenerative disc disease, low back pain

## Abstract

Both the diagnosis and treatment of pain are evolving, especially in interventional approaches. Diagnosis of low back pain combines old and new methodologies, in particular, it involves an expanded role for ultrasound. While low back pain is a common complaint, there are many etiologies to the condition which must be explored before a final diagnosis can be made and treatment planned. Tumors and infections are rarely involved in low back pain but should be ruled out in the initial phase itself since failing to address them early can have devastating consequences. Some invasive treatments seem promising in the management of low back pain.

Treating musculoskeletal pain with regenerative medicine, such as platelet-rich plasma, holds great promise. Autologous blood products are safe and may help stimulate the body’s own responses for regeneration. The so-called “orthobiologics” play a role in sports medicine and the treatment of musculoskeletal pain. Neuromodulation, especially spinal cord stimulation, is undergoing a renaissance with new waveforms, devices, and a greater albeit incomplete understanding of its mechanisms of action. Spinal cord stimulation is not a first-line therapy and not all patients or all back problems respond to this treatment. Nevertheless, the therapy can be safe, effective, and cost-effective with appropriate patient selection. Radiofrequency ablation of nerves in the form of neurotomy can be effective in reducing the pain of osteoarthritis. These procedures, including the newer cooled radiofrequency neurotomy, can restore function, reduce pain, and may potentially have an opioid-sparing effect. Technical expertise in nerve and anatomy is needed for the use of this technique. This review article aims to provide updated information on some invasive intervention techniques in pain management.

## Introduction and background

Prolonged, severe, or persistent pain can have a devastating impact on the quality of life of a patient. Novel therapeutic options in regenerative medicine and radiofrequency neurotomy offer new ways to treat pain associated with osteoarthritis and other conditions but require careful consideration on the part of the clinician and patient to achieve the desired result. Left untreated, pain syndromes can become centralized and result in chronic painful conditions that can defy easy treatment [1]. While opioids are effective pain relievers, their role in the long-term management of chronic pain is controversial at best [2]. Healthcare professionals currently have novel tools and innovative devices in their armamentarium that can be deployed to help treat pain and alleviate the pain in patients and lead more productive and better-quality lives.

This review constitutes a summary of a series of presentations on pain management delivered in April 2023 in Cancun, Mexico. It is based on insights derived from the clinical practice of the authors and supported by the literature. Many of these procedures described in this review are novel and have not undergone extensive literary review or been the subject of randomized clinical trials. This is a narrative review.

## Review

Diagnosis of low back pain

One of the most effective tools in getting a more precise diagnosis of the diffuse “low back pain” complaint is the chair, because it allows the patient to sit and discuss the condition in great detail with the physician. When diagnosing back pain, it is important to remember that while the patient’s self-report is crucial, the patient’s perception of pain etiology may not be accurate. It should be noted that patients with the same condition may experience pain differently. Preceding any clinical diagnosis, the patient should provide a detailed history, description of pain symptoms, pain characteristics, and identification of pain site(s). Low back pain is a prevalent condition with myriad different aspects that can influence its diagnosis and treatment.

The clinician has an arsenal of diagnostic tools including ultrasound (US) imaging, radiography, CT, MRI, scintigraphy, and others. Clinicians look for anatomical abnormalities, which may be static or dynamic; signs of inflammation; tumors; fractures; signs of degenerative diseases; and stenoses. Imaging technologies are important for the initial diagnosis or to confirm a presumed prior diagnosis and, in some countries, may be particularly important for potential legal documentation [3]. There are about 22 sources of spinal pain, of which eight are the most likely causes. Although there are 22 items, they may work at any of several levels and sometimes combine with others; that is, it is possible that multiple sources are at work in a single patient (Table 1) [3].

**Table 1:** Important areas of investigation when evaluating painful back symptoms

Potential source of spinal pain	Frequent source?
Facet joints	Yes
Side and intermediate branches	
Sympathetic plexus T-L	
Annulus fibrosus of the disc	Yes
End plates	Yes
Vertebral bodies	
Iliolumbar ligament	
Ligamenta flava	
Interspinous ligament	
Supraspinous ligament	
Intertransverse ligament	
Thoracolumbar fascia	
Intertransversarii (small muscles)	
Interspinal muscles	
Psoas muscle	
Quadratus lumborum	Yes
Multifidus muscle	Yes
Longissimus muscle	Yes
Iliocostalis muscle	Yes
Latissimus dorsal muscle	

Discogenic pain can be differentiated from facet joint pain because disc-related pain is more likely to be symmetrical or bilateral [3]. Disc degeneration can be asymptomatic, but may still contribute to facet joint arthropathy. Facet joints seem vulnerable to vertebral changes brought on by osteoarthritis. In many ways, the presentation of discogenic back pain is similar to that of pain in the vertebral column, which can arise from compression fractures, microfractures, and/or disc degeneration. Novel treatments which target the nerve supply of vertebral bodies show promise [3]. Muscles, fascia, or ligaments may be the source or a contributing factor to back pain, although myofascial sources of back pain are often labeled “nonspecific” [3].

Spinal stenosis, which in half of all cases is asymptomatic, can be subdivided into three types: central, foraminal, or lateral. Foraminal spinal stenosis is defined as a neuroforaminal diameter of <3 mm. Various forms of spinal stenosis may be caused by bulging discs, hypertrophic facet joints, epidural lipomatosis, or buckling or hypertrophy of the ligamentum flavum [3].

Imaging, in particular MRI, helps the clinician to align structural aberrations to pain reports, although imaging studies may be inconclusive [4]. In searching for the source of nociceptive pain in the spinal cord, radiological associations among anterior views (discs, vertebrae, columns), posterior views (facets, muscles, fascia, ligaments), and the spinal cord (stenosis, vasculopathy, possible myelopathy) can be helpful. The MRI may provide evidence of degeneration that does not cause or contribute to pain. A literature review reported that type I Modic changes, disc degeneration, deterioration of endplates, herniated discs, spinal cord stenosis, compressed nerves, and the infiltration of fat into muscle are likely to be related to low back pain [4]. Changes in bone marrow lesions or Modic changes in general (type I bone marrow edema and inflammation) may or may not (type II marrow ischemia and type III subchondral bone sclerosis) be associated with back pain [5]. When Modic changes cause painful symptoms, this is likely due to an inflammatory reaction in type I [4]. Facetogenic pain is involved in about 15% of all cases of low back pain, usually caused by inflammation of the zygapophysial joint [6].

Most nerves enter the vertebral body from the posterior via the basivertebral foramen. These nerves are mainly located in the vertebral center and branch out to their terminations in upper and lower endplates. The central vertebral endplates are densely innervated and contain nociceptors, making them a likely source of pain signals [7]. Edema around a vertebral endplate suggests pain. Pathological endplates are more likely than pathological discs to be densely innervated, but not all endplate conditions are clear on MRI [8]. Modic changes on MRI exhibit dense nerve networks with nociceptive fibers [8].

Case reports have demonstrated that patients who experience painful vertebral compression fractures may be experiencing pain from the posterior elements rather than the fractures themselves. Biomechanical models show that when a vertebral fracture causes a deformity, the vertebral column will compensate by cephalad or caudal subluxation. The compression fracture causes the anterior column to lose height, shifting the posterior column. This, in turn, means that the source of pain in such cases will come from posterior elements and, in such instances, pain can be controlled by medial branch blocks [9].

Degenerative disc disease

Disc degeneration has been classified by Pfirrman into five levels based on morphological degeneration: changes in disc height, annular tears, bulging discs, osteophyte formation, and intervertebral disc degeneration [10,11]. The severity of disc degeneration generally correlates with pain intensity. The Pfirrmann classification system is based on MRI signal intensity and has been shown to have good interobserver agreements and was robust when evaluating geriatric as well as younger subjects (Table 2) [10].

**Table 2:** The Pfirrmann classification system based on MRIs* *[12] NP: nucleus pulposus

Grade	Differentiation between the NP and the annulus	Signal intensity of the NP	Disc height
I	Clear differentiation	Homogeneous, hyperintensive	Normal
II	Clear differentiation	Hyperintensive with hypointensive horizontal banding	Normal
III	Blurred	A slight decrease in signal	Slightly decreased
IV	Lost	Moderate decrease in signal	Moderately decreased
V	Lost	Hypointense	Collapsed

Modic changes in vertebrae and endplates affect up to 350 million adults globally but their role in back pain remains unclear [13]. Modic changes are grouped into three distinct types (Table 3) [12].

**Table 3:** A summary of Modic changes in vertebrae and endplates based on T1- and T2-weighted images* *[12]

Type	T1	T2	Histopathology
1	Hypointense	Hyperintense	Bone marrow edema
2	Hyperintense	Hypointense	Fatty replacement
3	Hypointense	Hypointense	Sclerosis

Degenerating discs cause pain in different ways or may be asymptomatic. When degeneration causes the loss of mechanical function or damages the physiologic structure of the disc, this can reduce disc height, which, in turn, increases the risk of fissures in the annulus fibrosus and may set the stage for disc herniation. The loss of disc height can cause shifts in the spinal structures, adding stress to muscles, ligaments, and facet joints, which the patient would perceive as painful. Diminished disc height and/or disc herniation can also put undue and painful pressure on the nerve root that exits from the disc. Pain can occur as a direct result of the damaged disc due to inflammation and the overexpression of substance P [12,14]. Discs have very little innervation and limited vascular structure, but the growth of nociceptive nerves and vasculature can occur in a damaged disc and trigger back pain [12]. In some cases, a single-photon emission CT (SPECT-CT) scan may be helpful in the diagnosis. While a conventional CT scan uses radiographic energy to provide images, the SPECT-CT scans use a radiotracer to enhance the CT image.

When planning for surgery, the global alignment and proportion (GAP) score is a validated method to look at pelvic-incidence-based proportional parameters as means to predict mechanical problems. GAP analyzes the sagittal plane in adults with suspected spinal deformities [15]. Failures in sagittal alignment are a main cause of mechanical complications following surgery for spinal deformity and the GAP method and Schwab assessment tools have been proposed to help stratify risks and identify the proper surgical targets. In a head-to-head comparison, it was found that while both Schwab and GAP systems could reliably predict potential mechanical complications, GAP was statistically superior [16]. A GAP calculator tool is accessible online (gapcalcultator.com). The clinician enters individual data including age, pelvic incidence, sacral slope, L1-S1 lordosis, L4-S1 lordosis, and global title. This then calculates the patient’s individual GAP score by evaluating and weighting these factors, and a preoperative planning guide for the various regions of the spine with current angles and calculated “ideal” values. It should be noted that GAP calculations may not be appropriate for Asian patients due to different values in alignment compared to Europeans and others [17].

**Figure 1:**
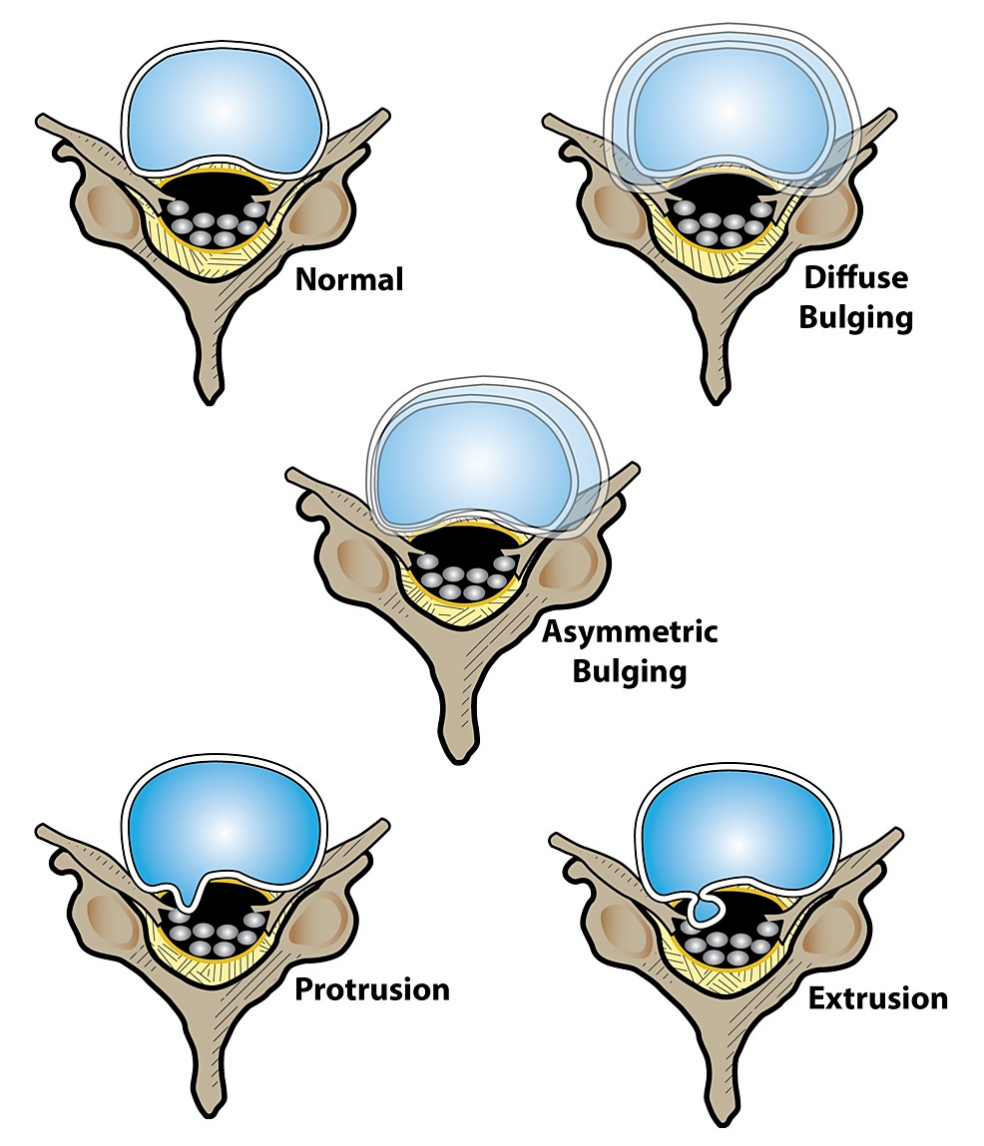
The main types of bulging discs Artwork by Todd Cooper of the Coyote Studios, Green Valley, California

**Figure 2:**
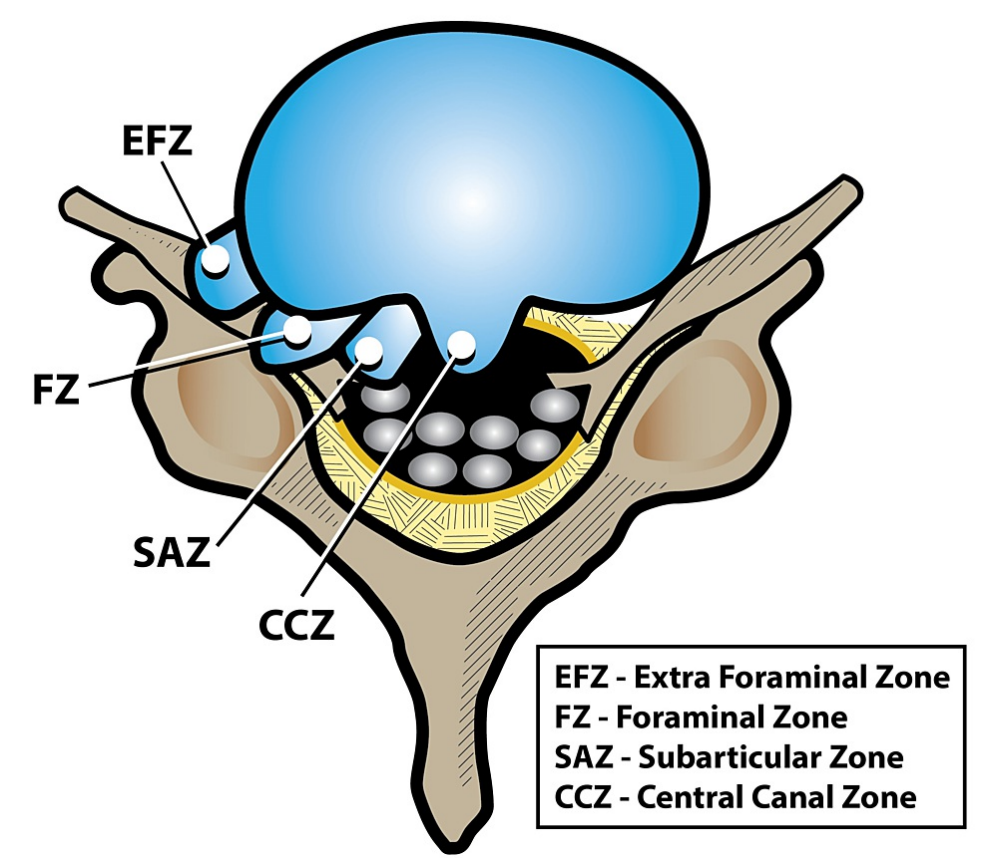
A disc bulging into the foraminal zone (FZ) can be intensely painful. Bulges into other areas may be associated with less pain or even be asymptomatic Artwork by Todd Cooper of the Coyote Studios, Green Valley, California

A bulging disc may bulge asymmetrically or the bulge may be diffuse, that is, relatively similar all the way around, while herniated disc may protrude or extrude. Disc damage that causes nerve compression around the foraminal zone is the most painful (Figures 1, 2). A bulge that affects the extraforaminal or subarticular zone may also be painful, but some patients are able to tolerate this pain and hence no intervention is required.

Annular fissures

**Figure 3:**
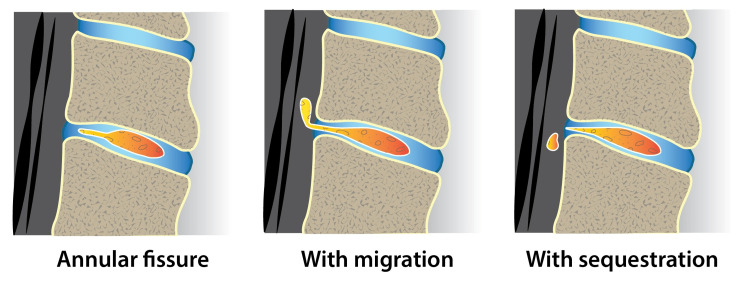
Annular fissures may have a migrated or sequestered morphology. They often do not require specific treatment Artwork by Todd Cooper of the Coyote Studios, Green Valley, California

Fissures in the annulus may be asymptomatic, can cause pain, and, in many instances, heal on their own (Figure 3). If a fissure is sequestered or if there is migration, it may compress the nerves and cause pain.

The primary cause of disc degeneration is lumbar disc herniation, which may be caused by obesity, occupational hazards such as manual labor, sedentary or unhealthful lifestyle, and/or aging. Lumbar disc herniation can press against the sinus nerve or nerve roots causing low back pain. In cases of radiculopathy, nerves going to the muscles may be affected. In many instances, patients with lumbar disc herniation will have low back pain as their presenting symptom with some pain or loss of sensation in the lower limbs [18]. A retrospective analysis of lumbar CT scans of patients with herniated lumbar discs (n=120) and controls (n=60) was analyzed, grouping the disc herniation patients into those who reported lower limb pain (n=60) and those with low back pain (n=60). Within the latter two groups, there was further stratification into three groups based on pain intensity. The analysis suggested that thinning and/or atrophy of the psoas muscle may be associated with lumbar disc herniation, with a greater correlation between psoas muscle atrophy and low back pain than lower limb pain [18].

Neurogenic claudication

Neurogenic claudication is caused by progressive degenerative changes to the spine, resulting in the compression of nerves in the lumbar region. It differs from vascular claudication where blood flow to the legs is impeded. Neurogenic claudication is associated with moderate to severe pain and may result in functional loss [19].

The patient’s position during MRI is important. In a study of 19 patients with low back pain or radicular pain that had not responded to conventional therapy or interventional treatment, buckling and increasing ligamentum flavum thickness were observed in 53% of cases where the patient was prone, but this was not seen in supine images. Facet joint subluxation and foraminal stenosis were seen in 26% of prone-position MRIs but not in supine images. Prone MRI is important in diagnosing this condition and may be helpful even in cases where the patient had previously undergone an inconclusive supine MRI [20].

Lumbar spinal stenosis

The progressive narrowing of the spinal canal compresses the dural sac, the spinal cord, the nerve root, or the cauda equina nerve and can result in clinical symptoms. Since it often starts with mild symptoms and progresses slowly, conservative therapies are usually employed first until severe pain and/or neurological deficits necessitate surgery. There are no clear-cut guidelines for the sequence of treatment, which may involve pharmacologic treatments, decompression, and fusion [21]. Evidence remains equivocal as to whether surgical treatment is superior to conservative management [22].

CT epidurography can be helpful in diagnosing the size and shape of spinal canal stenosis. A healthy spinal canal is round with a large dural sac and an epidural space that is close in size to the dural sac. With progressive stenosis, the dural sac decreases in size, with a greater difference between the dural sac and the epidural space. As it progresses, the stenosis may take on any of several morphologies, but eventually will completely block the spinal canal [23].

Tumors and infections

Positive electron tomography (PET) and CT imaging can be helpful for the identification of infections, such as osteomyelitis, diskitis, and spinal epidural abscesses, which can cause low back pain or contribute to back pain in patients with other back pain triggers. While vertebral osteomyelitis has a classic presentation of focal weakness, fever, and back pain, some patients present with only back pain, causing the infection to go undiagnosed [24]. In patients with active cancer, it is often assumed that back pain relates to a tumor, but this is not always the case. In all patients with back pain, it is important in an initial encounter to consider tumors and infections, since these are serious conditions that must be treated promptly [25].

Piriformis syndrome

Approximately 85% of sciatica is discogenic [26]. Piriformis syndrome is an infrequent cause of sciatica, albeit one of the main causes of non-discogenic sciatica. The flat piriformis muscle in the pelvic region can apply undue pressure on the sciatic nerve, causing inflammation, pain, and numbness, which may traverse the buttock and down the leg. Piriformis is likely due to a form of entrapment neuropathy [27]. Most patients respond to conservative treatments, but some patients need injections [28,29]. Magnetic resonance neurography plays a valuable role in identifying sciatic nerve anatomical variations, which may cause or contribute to nerve signaling systems and symptoms [30]. There are about six main configurations of the piriformis muscle and the sciatic nerve, and an understanding of sciatic nerve anatomy can be important to properly diagnose non-discogenic sciatica and avoid unintentional damage to this commonly encountered nerve during surgery [31,32].

Complex regional pain syndrome

Complex regional pain syndrome (CRPS) can be diagnosed using three-phase bone scintigraphy (TPBS). Notably, there can be significant differences in the blood pool phase on TPBS when comparing patients with CRPS and those without. On the other hand, when using digital infrared thermographic imaging (DITI), there was no significant difference and no way to readily distinguish those with or without CRPS [33]. The proper diagnosis of CRPS can be very challenging and must be based at least in part on clinical symptoms [34].

CRPS type I is characterized by pain and swelling of an extremity along with vasomotor deficits without evidence of a nerve injury. CRPS type II may present with the same symptoms but there is evidence of nerve injury. Should chronic pain develop in patients of either group, it will be primarily nociceptive for patients with CRPS type I, but neuropathic for those with CRPS type II. In real-world clinical practice, it is not unusual to see mixed pain develop. CRPS type III has been proposed to encompass forms of CRPS that do not fit well in types I and II [35]. Diagnosis can be complicated by the fact that a positive result on bone scintigraphy does not necessarily align with the presence of CRPS type I and a negative result does not always equate with the absence of CRPS type I [34].

Trigger points

Trigger or tender points are hyperirritable portions of the fascia or muscles. When pressure is applied to a trigger point, the patient may feel tenderness or pain. Trigger points may also trigger any number of other unpleasant symptoms such as fatigue, limited range of motion, and coordination problems. Experienced clinicians can locate trigger points during a physical examination in order to treat them with injections [36]. The puncture of trigger points to treat myofascial syndrome is most safely performed under ultrasound guidance in order to locate potentially hazardous anatomic structures that could impede the needle and allow for the accurate location of trigger points, even when those points are deep in muscle layers. Interfascial block performed under ultrasound guidance is a technique of regional anesthesia and may today be considered a promising technique for pain control [36].

Vibro-elastography is a cost-effective treatment that can be combined with ultrasound for trigger point localization. A vibro-elastography device combined with ultrasound can reveal trigger points as dark, non-elastic areas, which may then be treated by a dry-needling technique. Postural changes help to reveal the shear modulus on the ultrasound. Specifically, the shear modulus reduced after dry-needling in the prone compared to the sitting position, which aligns with their palpable degree of stiffness [37].

Considerations for the use of ultrasound in diagnosis

Damaged nerves may be detectable on ultrasound because of edema and larger surface areas. The cross-sectional area of a nerve (CSA) increases abnormally in neuropathic syndromes and fibromyalgia, which can be detected on ultrasound. In a study comparing the peripheral nerves in 50 patients with diabetes mellitus type 2 (T2DM) and suspected diabetic peripheral neuropathy to 50 age-matched control subjects, the CSA of peripheral nerves was sufficiently larger in the T2DM patients to be used as a morphological marker for diabetic peripheral neuropathy [38]. Likewise, the peripheral nerves of fibromyalgia patients have a higher CSA than controls, which is especially evident at the sural and vagus nerves and the sixth cervical nerve root [39]. Small fiber neuropathy appears to occur in about 40% to 49% of fibromyalgia patients, but diagnosing this condition can involve expensive procedures. Small fiber neuropathy has been associated with increased sural nerve CSA as well as obesity [40]. The CSA of the sural nerve as evaluated under ultrasound may be useful in diagnosing the subset of fibromyalgia patients with small fiber neuropathy [39,40].

Ultrasound can also aid in assessing osteoarthritis by showing cartilage and inflammation; it may be helpful in diagnosing plantar fasciitis, which can be further confirmed on elastographic examination if doubts still remain. Ultrasound can also be used with the local twitch response for myofascial pain.

Diagnostic wrap-up

An X-ray is usually the first image that is obtained, but ultrasound and other images may provide similar or better information. While MRI still has an important clinical role, it has sometimes been supplanted by other imaging tools. For example, in osteoarthritis of the hip, MRI has no clear-cut and validated biomarker. Since this type of imaging can be costly, may be contraindicated in some patients, and access to these machines may be limited, MRI may not always be used in real-world clinical practice or in clinical trials [41].

When using imaging techniques for a pain diagnosis, it is important to discuss the cases with the radiologist or radiology team. In general, requests should be made for only those tests that might provide a red flag that can change your diagnosis. Some diagnoses are complex and may require unusual approaches, but there should not be any hesitation in taking the appropriate steps to get the diagnosis.

The future of interventional pain care

Regenerative medicine is a recognized branch of bioengineering that utilizes a combination of cells, engineering methodologies, and biochemistry in order to improve or replace certain biological functions. In regenerative medicine, the body is compelled to use its own systems, sometimes with the help of exogenous biological materials, to recreate cells and reconstruct organs and tissues [42,43]. Regenerative medicine has an exciting new role in the treatment of musculoskeletal pain since these painful conditions potentially involve joints, tendons, ligaments, fascia, cartilage, vertebral discs, and nerves, all of which may benefit from regenerative approaches.

Biological products derived from the blood, such as platelet-rich plasma, are particularly useful as they have a natural antimicrobial effect, are safe for use, and can reduce localized inflammation while promoting cartilage and synovial anabolism. Platelets are anucleate fragments of cells that act like the “first responders” of the vasculature system; they immediately rush to repair breaches and restore homeostasis. Platelet-rich plasma has greater antimicrobial activity than platelet-poor plasma [44]. The platelet-rich plasma obtained from four patients with moderate to severe periodontal disease was collected and compared to the platelet-rich plasma of four subjects with healthy periodontium, and the study found that the platelet-rich plasma of all patients from both groups exhibited antimicrobial properties that would be effective against Porphyromonas gingivalis, an anaerobic bacteria recognized as the primary pathogen involved in periodontitis [45]. New research suggests that platelet-rich plasma can also stimulate immune cells to fight pathogens, but this can involve a delicate balance between pro- and anti-inflammatory responses [46].

Over the past 13 years, autologous biological products have been studied for their ability to aid in the treatment of skeletal muscle pathologies, such as osteoarthritis and tendinopathies. Both platelet-rich plasma and leukocyte-rich platelet-rich plasma are supported with good-quality evidence as a treatment for patellar tendinopathy and plantar fasciitis. However, evidence remains more equivocal for other applications, such as hip osteoarthritis, rotator cuff tendinopathy, and muscle injuries [47]. These so-called “orthobiologics” play an important role in sports medicine and modern orthopedics [48]. Autologous blood-derived products are increasingly used for the treatment of knee osteoarthritis, although evidence from many studies remains mixed. More precise classification of platelet-rich protein types may be needed to find the optimally effective product [49]. Platelet-rich plasma provides a concentrated mass of activated platelets, which, in turn, can deliver biomolecules directly to the lesion site. These biomolecules modulate the inflammatory process, promote angiogenesis, and boost the immune response, all of which aid in the repair of damaged tissue [50]. Platelet-rich plasma, whose autologous sourcing gives it an excellent safety profile, can be matched to clinical pathology and allows for minimally invasive delivery [50].

A particularly helpful classification system for defining platelet-rich plasma by Dohan Ehrenfest et al. used white blood cell content and fibrin architecture to create four different types of platelet-rich plasma, as shown in Table 4 [51]. Other classification systems use the number of platelets, the activation system, and the presence or absence of white blood cells [52]. No classification system or nomenclature is used universally, but it is essential that clinicians consider the characteristics of the platelet-rich plasma they select [53]. The specific factors important for platelet-rich plasma to be used to treat musculoskeletal disorders have not yet been fully elucidated [54].

**Table 4:** Summary of the classification system for platelet-rich plasma by Dohan Ehrenfest et al. It should be noted that this is not the only such system in use PRP: platelet-rich plasma; PRF: platelet-rich fibrin

Category	Definition	Applications
Pure PRP	No leukocytes, fibrin network is of low density	Suitable for injections
Leukocyte-rich PRP	High concentration of white blood cells, high concentration of platelets, fibrin network is of low density	Suitable for injections
Pure PRF	No leukocytes, fibrin network is of high density	Allow for a clot with growth factors
Leukocyte and PRF	High concentration of leukocytes, fibrin network is of high density	Allow for a clot with growth factors

There are many ways to prepare platelet-rich plasma, which can affect the characteristics of platelet-rich plasma, such as the rate at which it is extracted, which means that a needle gauge is important. The parameters of the centrifugal processes play a role. Other variables may be taken into consideration, which contributes to the heterogeneous nature of the various preparations in current use. This leads not only to different results but using platelet concentrations greater than 1800 X 103 platelets/µL can have adverse, even harmful effects. An excessive number of platelets can cause cellular apoptosis, downregulation, and de-sensitization of the growth-factor receptors, in turn resulting in paradoxical inhibition [52,55].

While it is established that regenerative medicine can change interventional pain care, it is not clear how to apply regenerative medicine in interventional procedures for chronic pain. Clinical trials often include only those patients whose pain derives from a single source, but in real-world clinical practice and particularly with chronic pain patients, there are multiple factors that contribute to the pain syndrome. For example, there are many potential pain generators associated with low back pain, necessitating a multifactorial approach. Multifactorial pain care now may include regenerative medicine plus conventional pharmacologic medicine.

It is crucial for clinicians to remember that more than one pain mechanism may be at work at a single pain site and different pain sites may have different mechanisms or combinations of them. For example, it is not unusual for a chronic pain patient to have both nociceptive and neuropathic pain in a single site. Furthermore, many musculoskeletal structures and systems are closely related, and pain or changes in one area can affect other areas. Careful examination of all pain sites is required and sequential diagnoses may be helpful. Clinicians should also consider the numerous risk factors for acute and chronic musculoskeletal pain: overweight, obesity, diabetes, sedentary lifestyle, carrying heavy burdens, mental health conditions, and certain forms of manual labor. The physical examination of a patient with chronic musculoskeletal pain should be thorough and systematic, including a neurological examination. Myofascial signs and trigger points should be considered here as potential peripheral pain generators [56].

Spinal fusion is a common technique, but it may not always deliver optimal results. The hardware in the body may create its own pressures. Also, structures can change with time, and hence an original fusion may have to be supplemented later with fusion at the next level. At one time, fusion was a cutting-edge approach, but today it is known that chronic lesions penetrating the subchondral bone may repair themselves if the production of fibrocartilage can be stimulated, although the repaired areas may be left with somewhat less mechanical strength than the original articular cartilage [57]. There have been promising results in treating these lesions - which are pain generators - with injections into the tissue of platelet-rich plasma under ultrasound guidance. These treatments are less traumatic for the patient than surgical interventions such as fusion.

Orthobiology, the science of biological restoration using regenerative techniques, has transformed the treatment paradigm for acute and chronic musculoskeletal pain. Orthobiology originated with hyaluronic acid supplementations, and then moved to platelet-rich plasma treatments and bone marrow concentrates. Today, studies are ongoing using cells derived from adipose tissue and the umbilical cord [58]. One can even summarize the stages of pain medicine in three main epochs with three keywords: remove, replace, and regenerate. This last stage is the most promising, least harmful, and hopefully will be soon used extensively.

Spinal neuromodulation

Neuromodulation encompasses a vast range of life-changing devices, ranging from Cochlear implants to restore hearing, deep brain stimulation (DBS) for Parkinson’s disease, sacral nerve stimulators for incontinence, and spinal cord stimulation to relieve chronic back pain. For every neuromodulatory device currently in use, there are many more in development. The impact of neuromodulation on medicine in general and pain control in particular cannot be overstated.

The ancient Romans used electrical energy from torpedo fish to treat gout pain, but it was only in the twentieth century and beyond that electrical power for medical purposes was fully harnessed. The first spinal cord stimulator became commercially available in 1968. Various incremental improvements were made over the following years, including an implantable system, burst stimulation options, and a closed-loop system [59,60]. Other advances include novel waveforms, artificial skin, implantable or injectable electrodes, and stimulation intended to restore functional loss [60].

The premise of spinal neuromodulators is that electrical energy applied to the nerves stimulates the nerves along the spinal cord and modifies their actions in such a way as to reduce painful stimuli from reaching the brain. Spinal neuromodulation can treat neuropathic as well as nociceptive pain and has even been effective in addressing the pain of complex regional pain syndrome. It can treat peripheral neuropathic pain syndromes, pain caused by ischemia, and even chronic visceral pain syndromes [61]. Although not a first-line approach, spinal cord neuromodulation in its many forms has found its place in routine pain medicine for certain conditions. In some ways, spinal neuromodulation may be seen as an intermediate step between pharmacologic treatment and surgical interventions. The main forms of spinal neuromodulation of interest to pain medicine are spinal cord stimulation, sacral nerve stimulation, dorsal root ganglion stimulation, occipital nerve stimulation, peripheral nerve stimulation, and peripheral nerve field stimulation [60].

While spinal cord stimulators are increasingly common and implant techniques well established, it is crucial to remember that these devices were never intended to operate as “set it and forget it.” When properly adjusted, spinal cord stimulation not only reduces aberrant pain signals reaching the brain, but it may also work to restore the normal pain inhibition pathways and, in that way, cause the body to produce its own endogenous pain-relieving neurotransmitters. Spinal cord stimulators can improve microcirculation in the spine as well. They must be periodically checked and parameters adjusted to deal with the patient’s changing condition, disease progression, and other factors.

Clinical considerations for the effective use of spinal cord stimulators are the proper selection of appropriate patients and matching them to the device best suited for their needs. While spinal cord stimulation is a powerful therapeutic tool, it does not work for all patients, and nor does it alleviate all types of pain. Furthermore, spinal cord stimulation may not be reimbursed by all payers, although cost-effectiveness analysis supports its use in many applications [62]. The reimbursement pattern varies among countries. Although spinal cord stimulation can be effective in treating the pain associated with critical limb ischemia, it may not be cost-effective in that particular application [63].

The mechanism of action of spinal cord stimulation is thought to be tonic stimulation or stimulation using a low frequency (40-80 Hz), a long pulse duration (200-500 µsec), and a high amplitude (3.5-8.5 mA). These waveform parameters allow a high charge to be delivered in each pulse, generating an action potential at the site of stimulation. This results in orthodromic activation of the large Aβ nerve fibers, producing paresthesia. This paresthesia at the pain site can produce effective pain control. The waveform parameters can be adjusted and the electrode placement modified using intraoperative and/or postoperative mapping to provide the appropriately placed “paresthesia zone.” The orthodromic activation can, in turn, activate the supraspinal pathways that can reduce pain; it can also result in antidromic activation of the Aβ fibers, which activates the inhibitory interneurons in the dorsal horn, which attenuate pain signals [64].

Not all patients respond to spinal cord stimulation, which led to the introduction of the sub-perception algorithm, modulating specific elements within the dorsal horn for effective pain control regardless of whether or not paresthesia occurs [65]. A new generation of spinal cord stimulators offers multiple programmable waveforms, including a tonic waveform (conventional), burst, 1.2 kHz for high-frequency stimulation, and dorsal-horn high-frequency stimulation [65,66]. High-frequency spinal cord stimulation is sometimes called paresthesia-free stimulation, although in some cases paresthesia still occurs. Most of these waveforms have high frequency (≥10 kHz), short pulse duration (30 µsec), and low amplitude (1-5 mA). Because paresthesia typically will not occur with these high-frequency pulses, there is no need for intraoperative mapping of paresthesia zones. Instead, electrodes are placed anatomically based on pain site locations. Comparative studies of low- versus high-frequency spinal cord stimulation have been favorable in that high-frequency works as well if not better than low-frequency stimulation [67-69]. An intriguing and promising idea being studied now is the potentially synergistic combination of high-frequency spinal cord stimulation plus conventional low-frequency stimulation.

Burst stimulation is another type of paresthesia-free stimulation designed to mimic a natural mode of information transmission in the spinal system. This method was developed by Dr. De Ridder who observed five monophasic spikes leading to a passive charge. Using a 40 Hz burst frequency and 1000 µsec pulse width, the burst creates a series of spikes at an intraburst rate of 500 Hz. Between bursts, there is a short pause or quiescent phase. Burst stimulation modulates the lateral and medial affective pain pathways [70-72].

The Differential Target Multiplexed™ (Medtronic, Minneapolis, MN) or DTM waveform is a proprietary waveform that disrupts the central nervous system processes mediated by the glia [73]. Chronic pain is caused and sustained by an imbalance between the neurons and the glia of the central nervous system. As metabolic and catabolic system regulators, glia plays an essential role in neural tissues and they vastly outnumber neurons. When the balance between glia and neurons is deranged, homeostasis at neural synapses can be lost. The DTM system uses different pulse signals for different types of cells; the term “multiplexed” is used to indicate that multiple types of signals may be combined with the delivered stimulation. The goal of this system is to bring the neuro-glial interactions back into balance by stimulation that targets neurons differently than glial cells. A typical waveform of this system has “multiplexed signals” with frequencies that might range from 500 to 1200 Hz and pulse widths that vary between 50 to 400 µsec. In clinical tests, differential target multiplexed spinal cord stimulation provided superior pain relief compared to conventional spinal cord stimulation in a 12-month study of patients with chronic low back pain [74].

The mechanisms of action of spinal cord stimulation remain incompletely elucidated and may be the reason that while these devices can provide effective relief of focal neuropathic pain, innovations have not always improved outcomes. It has been established that spinal cord stimulation provides effective analgesia in some patients, results in observable brain changes, and has been associated with neurochemical and physiologic changes in the spinal cord itself [65,75]. While spinal cord stimulation is based on the gate theory of pain, it is perplexing that it is more effective in treating neuropathic than nociceptive pain [75]. Recent research suggests that dorsal column stimulation may be the mechanism of action but the post-synaptic dorsal column pathways suggest that this stimulation may be indirect. This, in turn, has led to the speculation that dorsal horn stimulation via dorsal horn islet cells may be the primary site of action [75].

In some ways, a pharmacologic analgesic model can be helpful in understanding spinal cord stimulation in that there is a defined therapeutic window, above which is toxicity and below which is a sub-therapeutic zone. The distance between the electrode tip and the targeted area of the spine affects the amount of charge delivered. This is particularly true of epidural electrodes where the dural thickness and dorsal layer of the cerebrospinal fluid must be considered. It has been observed that physiologic movements such as respirations and cardiac activity as well as postural changes can result in large changes to the amount of charge delivered and impact therapy [76].

Guidelines from the National Institute of Health and Care Excellence (NICE) state that spinal cord stimulation can be safe, effective, and cost-effective for patients suffering from severe refractory neuropathic pain, but these devices are not often used in this specific population [77]. Spinal cord stimulation is not a first-line treatment but is advised only after pain has been diagnosed as refractory neuropathic pain and the patient has had this pain for at least a year with no significant improvements using conventional treatments [77]. Beyond the selection of appropriate patients, patients should be educated for a better understanding of pain management techniques and supported as they trial and adjust to a spinal cord stimulator. Using mutual decision-making paradigms, clinicians and patients should work together to allow the patients to reduce the use of opioids and other analgesics, employ new pain management techniques such as exercise or relaxation, and regain functional losses.

Psychological factors should be considered in patient selection, although the association remains unstudied and unproven between mental health conditions, such as depression or anxiety, and poor outcomes with spinal cord stimulation [78]. The literature provides evidence to support that certain psychological factors, such as depression, anxiety, somatization, catastrophizing, and poor coping skills, may be associated with suboptimal analgesic therapy in general [79]. Patients should be coached with realistic expectations so that they are not disappointed if spinal cord stimulation does not eradicate their pain completely.

Trialing spinal cord stimulation is often a required clinical step, but there is no high-quality evidence from randomized clinical trials to demonstrate that a trial period predicts long-term outcomes [80]. Nor is it known how long a trial should last and under what conditions it should be conducted. In general, the trial allows patients to experience what spinal cord stimulation feels like, to determine if and how it may affect pain, and to give the clinical team ideas regarding optimal lead placement and device parameter settings. In general, success means that the device is able to reduce pain by at least 50%. Trial conditions should be tailored for various painful conditions [81]. There are important pitfalls that can occur. Percutaneous electrodes can migrate during a three-day trial [82]. The device can become infected. Patients may dislike the inconvenience of the trial. Evidence from the TRIAL-STIM clinical study suggests that patients prefer to dispense with the trial phase altogether. The reasons patients prefer to avoid the trial period are as follows: patient convenience, less time off work, no hassles with “loose wires,” and having only one recovery period [80]. Regulations about the necessity of a trial phase vary among countries.

Patients who trial paresthesia-free spinal cord stimulation have very little sensory feedback and may be unable to detect changes in baseline pain. Patients whose trial includes only one waveform may not get adequate results because not all waveforms are effective for all types of pain and patients. When multiple waveforms are available, the trial should test different variations. The duration of the trial is problematic, because the time needed to adequately test spinal cord stimulation may be very long that the temporary implant can become infected. In a study of 40 patients who had a trial of a spinal cord stimulator, there was a 7.5% infection rate [83]. 

Many chronic pain patients are willing to advance to permanent implants. Complications with spinal cord stimulators are most likely to occur in the initial year post-implant and these complications are rarely serious or fatal [84]. Nevertheless, complications have been reported in a literature review to occur at a rate of 34% [85]. Technical complications can occur with any device-based treatment, and with spinal cord stimulators, there is a possibility of lead fracture, lead malfunction, and lead migration. There can also be problems with the stimulator such as very rare random component failures and battery problems. Technical problems may occur in the perioperative period or within about the first two years after implant [86]. Devices may need to be adjusted periodically, which is not a complication but may represent an inconvenience to the patient. Some patients complain about pain or seroma over the device. Dural punctures have also been reported [86].

Wearable sensors, artificial skin, and innovative new waveforms and device settings are likely to be available soon. The electrode surface area that comes into contact with the body tissue is a main focus for innovation. The goal is to develop a small, very durable yet highly sensitive electrode that will interface with the body without damaging tissue. Optimal electrode performance may require low impedance values, which can be produced with specific materials and a large surface area of the electrode, but low-impedance electrodes may be too large for the delicate areas within the spine. In addition, electrodes for use in spinal cord stimulation must resist flexing, bending, or kinking. All of these are areas of current innovation and testing. Lead materials include platinum and iridium alloys, platinum alone, titanium, and gold, which must resist cracks and fissures. Conductive polymers are being explored as a way of helping to minimize the mismatch between electrodes and tissues and providing low impedance values with small surface areas [87,88]. Injectable electrodes have also been developed, which help to form a neural electrode in the body [89].

Radiofrequency neurotomy for osteoarthritis patients

Osteoarthritis is a progressive and painful degenerative disease that reduces joint cartilage and erodes the articular surface [90]. As life expectancy increases, clinicians are likely to encounter more patients with osteoarthritis in the future. The main symptom of osteoarthritis is pain, which can impose functional limitations, fatigue, depression, and a loss of independence. Up to a quarter of people with osteoarthritis will lose their ability to live independently [91].

Current treatment of osteoarthritis involves attempts to halt or at least delay the destruction of joint cartilage and to improve the patient’s quality of life [92]. There are a number of nonsurgical approaches that have been shown to effectively alleviate pain and reverse the disease [93,94]. These therapies include physiotherapy, weight loss, oral nonsteroidal anti-inflammatory drugs (NSAIDs), interarticular steroid injections, hyaluronic acid injections, platelet-rich plasma, and shock waves [95,96]. In particular, obesity is correlated with osteoarthritis and can have a profound impact on the disease. In a single-blind study of 24 community-dwelling older obese adults with knee pain, weight loss achieved through a diet and exercise program sustained over six months both significantly reduced pain and improved performance [97].

Advanced osteoarthritis may be successfully treated with arthroscopic surgery or arthroplasty [98]. Nevertheless, persistent pain may occur in up to 20-28% of patients after surgery, and many patients are not appropriate candidates for surgery in the first place because of their overall health and condition and/or comorbidities [99,100]. Radiofrequency ablation has emerged as a major focal point for current studies and the effective treatment of knee and hip osteoarthritis [101]. A meta-analysis of randomized controlled trials for knee osteoarthritis patients conducted in eight countries analyzed 15 studies (n=1,009 patients) published from 2011 to 2021. It found that radiofrequency neurotomy was safe and effective at relieving pain associated with knee osteoarthritis and improving function [102]. In this meta-analysis, eight studies applied radiofrequency energy to the genicular nerve while seven used an intra-articular application [102]. This meta-analysis concluded that radiofrequency was safe and effective for relief of the pain and recovery of the functional loss associated with osteoarthritis of the knee.

Radiofrequency ablation works by generating sufficient heat to cause thermocoagulation and the localized destruction of neuronal tissue. The threshold value for heat sufficient to destroy neuronal tissue is approximately 45 °C (113 °F) [103,104]. It is hypothesized that radiofrequency energy destroys the peripheral nociceptive inputs (Aδ fibers and C fibers) without affecting the motor and sensory fibers (A and B fibers). It is believed that radiofrequency ablation conserves the basal lamina of the Schwann cells, which allows the nerve to regrow [104].

An important clinical trial, in which radiofrequency neurotomy was used to treat the articular nerve branches of the genicular nerves, by Choi and colleagues provides good procedural guidance [105]. In this study, radiofrequency neurotomy was performed under fluoroscopy, significantly reducing pain, and restoring lost function, and with no adverse events following the procedure [105]. More recent studies have reported on more complete neurotomies. Cooled ablation devices and their techniques have also been reported [106]. Prognostic nerve blocks can help predict procedural outcomes, but they did not improve the procedural success rate [106]. A careful review of the nerves surrounding the knee joint and technical procedural considerations are necessary [107,108]

Radiofrequency neurotomy for knee pain is a safe procedure, but certain complications, such as infection and pain, have been reported. This procedure is indicated for treating moderate to severe osteoarthritis knee pain as well as for patients who were recently fitted with a prosthetic. Further studies are needed to see if radiofrequency neurotomy for knee pain could reduce the use of opioid analgesics, decrease pain intensity, and improve function [106].

## Conclusions

The diagnosis and treatment of musculoskeletal pain, particularly chronic painful conditions such as knee osteoarthritis, are being transformed with a greater elucidation of pain mechanisms, advanced imaging and diagnostic tools, and the use of regenerative and radiofrequency procedures. Pain can and should be treated, but it is important to match the appropriate patient with the most suitable type(s) of treatment, bearing in mind that multi-mechanistic approaches are sometimes required.
